# Comparison of Biofilm Formation Capacities of Two Clinical Isolates of Staphylococcus Epidermidis with and without icaA and icaD Genes on Intraocular Lenses

**DOI:** 10.4274/tjo.79059

**Published:** 2017-04-01

**Authors:** Sertaç Argun Kıvanç, Merih Kıvanç, Volkan Kılıç, Gülay Güllülü, Ahmet Tuncer Özmen

**Affiliations:** 1 Uludağ University Faculty of Medicine, Department of Ophthalmology, Bursa, Turkey; 2 Anadolu University Faculty of Science, Department of Biology, Eskişehir, Turkey; 3 Armedica Eye Center, Ophthalmology Clinic, Darıca, Turkey

**Keywords:** Biofilm, intraocular lenses, Staphylococcus epidermidis, hydrophobic, hydrophilic

## Abstract

**Objectives:**

To compare biofilm formations of two *Staphylococcus epidermidis (S. epidermidis)* isolates with known biofilm formation capacities on four different intraocular lenses (IOL) that have not been studied before.

**Materials and Methods:**

Two isolates obtained from ocular surfaces and identified in previous studies and stored at -86 °C in 15% glycerol in the microbiology laboratory of the Anadolu University Department of Biology were purified and used in the study. The isolates were *S. epidermidis* KA 15.8 (ICA+), a known biofilm producer isolate positive for *icaA, icaD* and *bap* genes, and *S. epidermidis* KA 14.5 (ICA-), known as a non-biofilm producer isolate negative for *icaA, icaD* and *bap* genes. The biofilm formation capacities of the 2 isolates on 4 different IOLs were compared. Two of the IOLs were acrylic (UD613 [IOL A], Turkey; SA60AT [IOL B], USA), and the other two were polymethyl methacrylate (PMMA) (B60130C [IOL C], India; B55125C [IOL D], India). Bacterial enumeration and optical density measurements were done from biofilms that formed on the IOLs. Biofilms were imaged using scanning electron microscopy.

**Results:**

Mean bacterial counts on the IOLs were 7.1±0.4 log_10_ CFU/mL with the ICA+ isolate, and 6.7±0.8 log_10_ CFU/mL with the ICA- isolate; there were no statistically significant differences. Biofilm formation was lower with acrylic lenses than PMMA lenses with both isolates (p=0.009 and p=0.013). The highest biofilm production was obtained on IOL C (PMMA) (p<0.001) and the lowest was obtained on IOL A (hydrophilic acrylic) (p<0.001).

**Conclusion:**

Bacterial counts after biofilm formation were lower on acrylic lenses, especially hydrophilic acrylic with hydrophobic properties. Further animal and *in vivo* studies are required to support the findings of this study.

## INTRODUCTION

**Figure 1:**
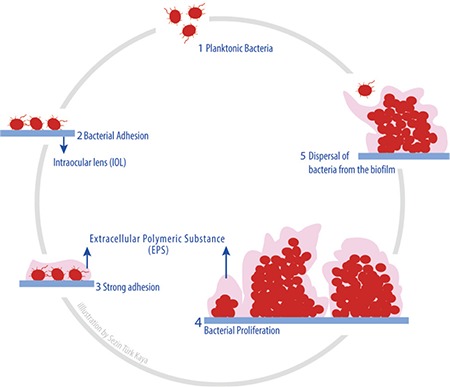
Schematic of biofilm formation on the intraocular lens surface

Postoperative endophthalmitis is one of the most serious complications of intraocular lens (IOL) implantation after cataract surgery.^1^ Various studies have reported postoperative endophthalmitis after cataract surgery at rates of 0.02% to 0.2%.^2,3,4,5,6^ *Staphylococcus epidermidis* is a coagulase-negative staphylococcus (CNS) and is one of the bacteria frequently isolated in postoperative endophthalmitis. CNS are a normal part of the flora of the eye and surrounding tissues.^7,8^ Surgical instruments or contaminated IOLs may introduce these microorganisms into the eye during surgery.^9,10^ Biofilms formed by bacteria have also been documented on ocular materials such as contact lenses, IOLs, glaucoma tubes, and corneal sutures.^11,12^ Previous studies have reported that *S. epidermidis* produces biofilms on IOLs.^9,13,14,15^ The formation of biofilms by *S. epidermidis* is dependent on microbial and environmental factors. The main microbial factor is whether the bacteria possess an *icaADBC* gene locus. Polysaccharide intercellular adhesin (PIA) is responsible for biofilm production in *S. epidermidis*.^16^ The ica operon synthesizes poly-N-acetyl-beta-1-6-glucosamine, which enables the formation of PIA. The ica genes allow *S. epidermidis* to synthesize a polysaccharide substance called the ß-1-6-glycosaminoglycan chain. Of the *ica* genes, *icaA* and *icaD* are more important in *S. epidermidis* biofilm production, allowing synthesis of sugar oligomers using UDP-N-acetylglucosamine as a substrate. This is how the biofilm begins to form (Figure 1).^10^ Biofilm formation is a complex process, and environmental factors are also important. The condition and chemical structure of the biomaterial surface, as well as properties such as hydrophilicity or hydrophobicity also play a key role.^13^ In this study, we investigated the biofilm production characteristics of two clinical *S. epidermidis* strains, one positive and one negative for the biofilm-producing *icaA* and *icaD* genes, on two acrylic and two polymethyl methacrylate (PMMA) IOLs that have not been previously compared.

## MATERIALS AND METHODS

### Bacteria

*S. epidermidis* cultures isolated from the ocular surface and purified in previous studies were stored at -86 oC in 15% glycerol. *S. epidermidis* KA 15.8 (ICA+: *icaA, icaD,* and *bap* (biofilm-associated protein) gene positive, high biofilm producing) and *S. epidermidis* KA 14.5 (ICA-: *icaA, icaD,* and *bap* gene negative) isolates were obtained from the Microbiology Laboratory of the Anadolu University Faculty of Sciences, Biology Department. The cultures were revived and their purity and viability checked before being used in the study.

### Intraocular Lenses

Four different IOLs were used. Two were foldable acrylic lenses: Acriva UD613 VSY, Turkey (IOL A) and AcrySof SA60AT, Alcon, USA (IOL B). The other two were PMMA: B60130C, Biotech, India (IOL C) and B55125C, Biotech, India (IOL D). IOL A is a hydrophilic acrylic lens with hydrophobic properties and 25% water content. IOL B is a hydrophobic acrylic lens with ≤2% water content. IOL C is a hydrophobic PMMA lens with ≤1% water content and two positioning holes. IOL D is a hydrophobic PMMA lens with ≤1% water content but without the positioning holes found in IOL C. The features of the lenses used in this study are shown in Table 1.

### Identification of Biofilms on the Intraocular Lenses

*S. epidermidis* was cultivated in Tryptic Soy Broth (TSB) containing 0.25% glucose for 24 hours at 37 oC. The IOLs were placed in 12-well polystyrene microplates (Griener, Turkey) with one IOL per well. The *S. epidermidis* cultures were diluted 1:40 with TSB containing 0.25% glucose, then 1 mL aliquots of the diluted cultures were applied to the IOLs in the plates. All plates were incubated at 37 oC for 24 hours. One group of IOLs was incubated in bacteria-free medium. After the incubation period, the presence of biofilm on the IOLs was assessed by spectrophotometry. Before measurement, IOLs were removed from the medium and washed 3 times with phosphate-buffered saline (PBS) and placed in a sterile plate. After drying, the IOLs were stained in 1% crystal violet for 15 minutes, then washed again with PBS. Finally, 200 μL of ethanol/acetone (80:20 vol/vol) solution was added to the IOLs to release the cells. These solutions were transferred to a multi-well plate and the optical density (OD) at 620 nm was read using a microplate reader.^17^

Biofilm production in the polystyrene wells was used as a control group. In each group, five trials were done in parallel.

### Enumeration of Intraocular Lens-Adherent Bacteria

IOLs cultivated as described above were washed with PBS, then each IOL was transferred to a 1.5 mL microtube containing 1.0 mm glass beads and 1 mL PBS was added. The tubes were vortexed for 1.5 minutes at 2500 rpm in order to separate the cells from the biofilm matrix. Dilutions were prepared and bacterial enumeration was done by drop plate method. All studies were done in five parallel trials.

### Scanning Electron Microscope (SEM) Analysis

Bacterial adhesion was examined by SEM as described by Okajima et al.^13^ with some modifications. *S. epidermidis* isolates were incubated in TSB containing 0.25% glucose for 24 hours at 37 oC. After incubation, the IOLs were carefully washed 3 times with PBS. The IOLs were fixed for 2 hours in room temperature 0.1 M phosphate buffered (pH 7.4) 2.5% (wt/vol) glutaraldehyde, then washed 3 times in 0.5 M sodium cacodylate for 15 minutes. After this process, the lenses were rinsed in distilled water and dehydrated using an ethanol series (50%, 70%, 80%, and 95%). After incubating at each concentration in the series for 7 minutes, the lenses were incubated in pure ethanol for 15 minutes. Immediately following the ethanol series, the drying procedure was performed in the Critical Point Dryer. The IOLs were then coated with gold and analyzed using SEM.

### Statistical Analysis

Statistical Package for the Social Sciences version 22.0 (IBM Corp., USA) software was used for all statistical analyses. T-test was used to compare bacterial counts and values obtained from the two strains with acrylic and PMMA lenses; Mann-Whitney U test was used for all other comparisons within each lens type. P values less than 0.05 were considered significant.

## RESULTS

**Figure 2:**
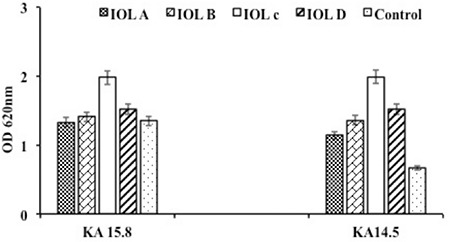
Crystal violet staining of the biofilms produced by S. epidermidis KA 15.8 (biofilm producer) and S. epidermidis KA 14.5 (non-biofilm producer) isolates on the different intraocular lenses IOL: Intraocular lens, OD: Optical density

We investigated the biofilm formation of one biofilm-producing and one non-biofilm-producing strain of *S. epidermidis* on acrylic and PMMA lenses. Biofilm evaluation by crystal violet staining and spectrophotometry revealed that both isolates formed biofilms to varying degrees on the IOLs (Figure 2). The known biofilm-producing ICA+ strain had a mean bacterial count of 7.1±0.4 log_10_ CFU/mL and mean OD value of 1.6±0.8 across all lens types. These values were 6.7±0.8 log_10_ CFU/mL and 1.5±0.3 for the ICA- strain. Although the values of the ICA+ strain were higher, the difference was not statistically significant. Bacterial count correlated with OD (p<0.001, r=0.720).

Although in theory the ICA- *S. epidermidis* strain is considered a non-biofilm-producer, we found that this strain also formed biofilms on the lenses. A comparison of the biofilm production characteristics of the ICA+ and ICA- strains on the lenses (acrylic and PMMA) is shown in Table 2. The ICA+ strain produced higher bacterial counts than ICA- strain on acrylic lenses, though the difference was not statistically significant. On PMMA lenses, both strains yielded similar results (Table 2). Statistical comparison of acrylic and PMMA lenses showed that there was less biofilm production on acrylic lenses compared to PMMA lenses in both strains (p=0.009 and p=0.013).

The highest biofilm production from both strains was seen in IOL C, one of the PMMA lenses. The least biofilm production occurred on IOL A, one of the acrylic lenses. Except for IOL A, bacterial counts were similar in the biofilms produced by both strains. The bacterial count on IOL A was significantly lower than on IOLs B, C, and D (p<0.001, p<0.001, and p=0.006, respectively). On IOL A, the bacterial count in the biofilm produced by the ICA- strain was significantly lower than that of the ICA+ strain (p=0.003). The bacterial counts and OD values of the biofilms formed on the IOLs by the ICA+ and ICA- strains are shown in Table 3.

**Figure 3:**
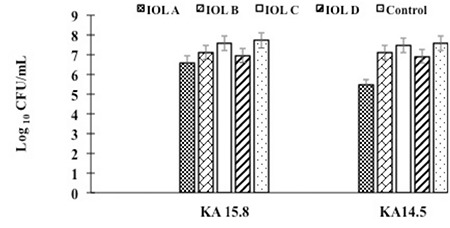
Bacterial counts in the biofilms produced on the intraocular lenses IOL: Intraocular lens

**Figure 4:**
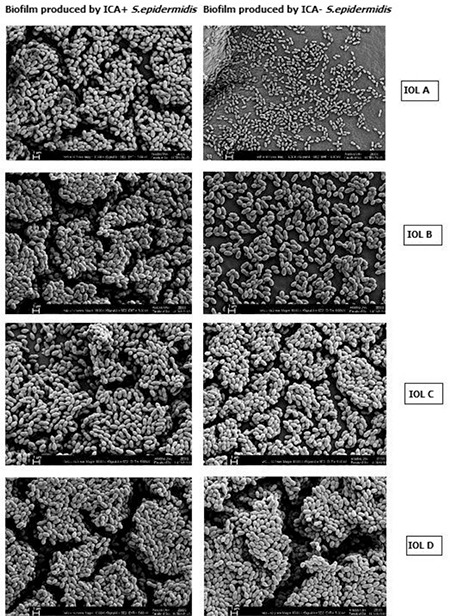
Scanning electron microscope images of the biofilms produced by S. epidermidis KA 15.8 (biofilm producer) and S. epidermidis KA 14.5 (non-biofilm producer) isolates on the different intraocular lenses IOL: Intraocular lens

Enumeration of bacterial colonies revealed high counts for both strains on all the lenses (Figure 3). Bacterial counts on acrylic lenses were lower compared with the other IOLs. Bacterial adhesion was observed via electron microscopy (Figure 4). In the SEM images of the biofilms produced by ICA+ *S. epidermidis*, a multi-layer structure was evident with all of the lenses. In contrast, in images from the ICA- strain, this multi-layer structure did not appear on acrylic IOLs, but was evident on PMMA IOLs.

## DISCUSSION

Biofilm formation enhances the virulence of bacteria and is a feature that confers resistance against antimicrobial agents.^12,18,19,20^ Biofilm production first began to draw the attention of the ophthalmology community at the beginning of the 21^st^ century and has steadily continued to gain importance. It was first documented in ophthalmology in 2003 by Kodjikian et al.,^21^ who obtained SEM images of ^S. epidermidis^ forming a biofilm on silicone IOLs with PMMA haptics and reported that strains carrying the ica locus produce biofilms more readily. The same authors reported in subsequent studies that bacteria type, incubation time, and IOL design influenced bacterial adhesion, but determined that the most important factor was lens material, and especially its hydrophobicity or hydrophilicity.^14^ In the current study, we also investigated the biofilm-forming properties of both *icaA*-positive and *icaA*-negative clinical isolates on PMMA and acrylic lenses.

In our study, both the strains with and the strains without *icaA, icaD*, and *bap* genes formed biofilms in both lens groups. We observed from the SEM images and during bacterial enumeration that the biofilm was formed in multiple layers. The two strains yielded similar results in both spectrophotometry and bacterial enumeration. Biofilm production in the strain negative for *icaA, icaD*, and *bap* genes may be attributable to the influence of virulence factors other than those gene loci in biofilm formation. Prasad et al.^22^ also reported that both **icaA**-positive and *icaA*-negative strains formed biofilms on PMMA lenses, which they confirmed through bacterial enumeration. In a similar study comparing biofilm-producing and non-producing *S. epidermidis* strains, Okajima et al.^13^ found that both formed biofilms intensely on acrylic lenses.

In our comparison of acrylic and PMMA lenses, we detected significantly more bacteria from both strains on the PMMA lenses. The many studies conducted on this topic have conflicting results. Okajima et al.^13^ reported the least *S. epidermidis* biofilm formation on silicone lenses and the most on acrylic lenses, although there was no statistical difference between acrylic and PMMA lenses. Schroeder et al.^23^ observed no differences between acrylic, silicone, and PMMA lenses. Baillif et al.^24^ found that bacterial growth over time was less on hydrophilic acrylic lenses compared to PMMA, hydrophobic acrylic, and silicone lenses. In contrast to these studies, Fazly Bazzaz et al.^25^ observed less biofilm formation on PMMA lenses compared to hydrophilic acrylic lenses. Many authors have suggested that biofilm production may be as dependent on microorganismal characteristics as it is on factors like lens material and surface properties.^10,14^ The different results obtained in the abovementioned studies may stem from variations in these characteristics. Those studies were all conducted using IOLs with different properties, not with a standard IOL. Furthermore, the characteristics of the isolated microorganisms also differed. Therefore, we believe comparing the results of these studies may lead to inaccurate conclusions. In the present study, we used two different acrylic lenses from different brands and two PMMA lenses with different properties from the same brand. One of the interesting results of our study is that the lowest bacterial colonization occurred on IOL A. This hydrophilic lens had the highest water content (25%) of all the lenses used in our study and is claimed to show hydrophobic surface behavior due to the lens’ unique composition. We also found a difference in bacterial count between the two different PMMA lenses of the same brand (IOL C and IOL D). IOL C has a larger surface area compared to IOL D because of its lens size, and it also has two positioning holes. We believe that these two factors make it easier for bacteria to colonize IOL C.

In cataract surgery, the IOL may become contaminated with bacteria before, during, or after implantation.^23,26,27^ The use of cartridges in IOL placement and even the introduction of ready, pre-loaded IOL cartridges have greatly reduced the probability of contamination before and during implantation.^28^ However, recent studies have reported that bacteria can be found on the ocular surface and the intraocular space due to influx at the end of cataract surgery performed using phacoemulsification in sterile conditions.^26,29^ It has been demonstrated that coating lenses with the inflammatory mediator fibronectin, which is activated during surgery, facilitates bacterial adhesion.^23^ For this reason, it is believed that solutions which alter a material’s surface can increase the biocompatibility of lenses. Schroeder et al.^23^ reported that *S. epidermidis* adherence was less on IOLs with surface modification. Nomura et al.^30^ found that heparinization of biomaterial surfaces reduced biofilm formation. Another study demonstrated that hydrophilic coating of a silicone material reduced microorganismal colonization.^31^ Various studies have shown reduced bacterial adhesion with lens coating and surface modifications.^12,23,32^ We do not know whether the result we obtained from IOL A is related to the hydrophilicity of the lens or the surface properties conferred by the molecular structure of the new material. We were unable to acquire information about the manufacturer’s lens production and the exact process involved.

## CONCLUSION

In summary, studies have identified a host of factors that influence biofilm formation. In the present study, the lowest bacterial counts were found on a hydrophilic acrylic lens with hydrophobic properties. This research must be supported with animal and in vitro studies. Biofilm formation has been observed in all studies conducted to date, and the production of an IOL completely resistant to bacterial adhesion is not yet a possibility. Recent studies suggest that antibiotics may be effective before biofilm formation, but the efficacy of antibiotics is limited after a biofilm has developed.^20^ Therefore, we believe that developing methods for the prevention of biofilm formation is more important than developing ways to treat patients with biofilms. Given these considerations, we believe that new strategies must be developed in lens production.

## Figures and Tables

**Table 1:**
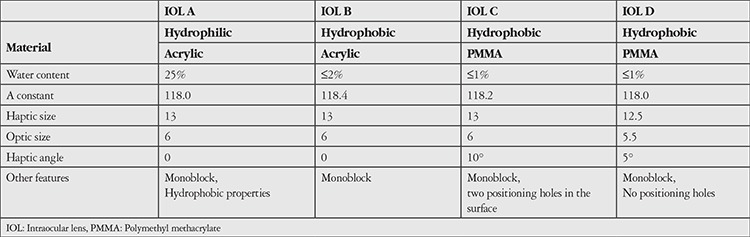
Properties of the intraocular lenses used in the study

**Table 2:**
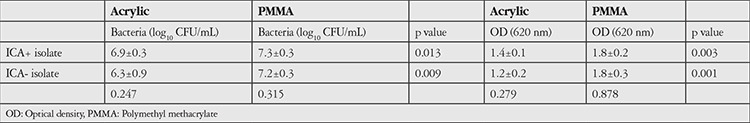
Bacterial counts and optical density values for both isolates on the acrylic and polymethyl methacrylate lenses

**Table 3:**
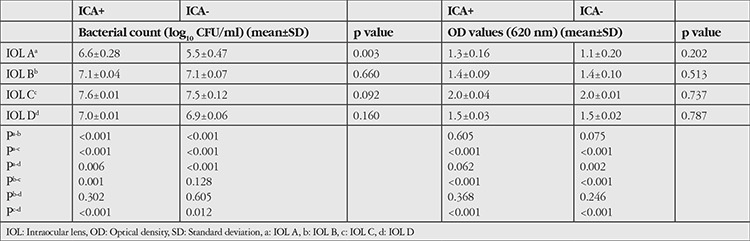
Comparison of bacterial counts and optical density values of the biofilms produced by both isolates on the 4 different intraocular lenses

## References

[1] Wong TY, Chee SP (2004). The epidemiology of acute endophthalmitis after cataract surgery in an Asian population. Ophthalmology..

[2] Taban M, Behrens A, Newcomb RL, Nobe MY, Saedi G, Sweet PM, McDonnell PJ (2005). Acute endophthalmitis following cataract surgery: a systematic review of the literature. Arch Ophthalmol..

[3] West ES, Behrens A, McDonnell PJ, Tielsch JM, Schein OD (2005). The incidence of endophthalmitis after cataract surgery among the U.S. Medicare population increased between 1994 and 2001. Ophthalmol..

[4] Miller JJ, Scott IU, Flynn HW, Smiddy WE, Newton J, Miller D (2005). Acute-onset endophthalmitis after cataract surgery (2000-2004): incidence, clinical settings, and visual acuity outcomes after treatment. Am J Ophthalmol..

[5] Jabbarvand M, Hashemian H, Khodaparast M, Jouhari M, Tabatabaei A, Rezaei S (2016). Endophthalmitis Occurring after Cataract Surgery: Outcomes of More Than 480 000 Cataract Surgeries, Epidemiologic Features, and Risk Factors. Ophthalmology..

[6] Esteban J, Pe´rez-Tanoira R, Pe´rez-Jorge-Peremarch C, Go´mez-Barrena E, Kon K, Rai M (2014). Bacterial Adherence to Biomaterials Used in Surgical Procedures. Microbiology for surgical infections Diagnosis, prognosis and treatment.

[7] Perkins RE, Kundsin RB, Pratt MV, Abrahamsen I, Leibowitz HM (1975). Bacteriology of normal and infected conjunctiva. J Clin Microbiol..

[8] Speaker MG, Milch FA, Shah MK, Eisner W, Kreiswirth BN (1991). Role of external bacterial flora in the pathogenesis of acute postoperative endophthalmitis. Ophthalmology..

[9] Schauersberger J, Amon M, Aichinger D, Georgopoulos A (2003). Bacterial adhesion to rigid and foldable posterior chamber intraocular lenses: in vitro study. J Cataract Refract Surg..

[10] Ahmad S, Ashraf H, S M Akram SM, Méndez-Vilas A (2015). Adhesion of Biofilm forming Staphylococcus epidermidis strains on Intraocular Lenses – An Update. The Battle Against Microbial Pathogens: Basic Science, Technological Advances and Educational Programs.

[11] Bispo PJ, Haas W, Gilmore MS (2015). Biofilms in infections of the eye. Pathogens..

[12] Zegans ME, Becker HI, Budzik J, O’Toole G (2002). The role of bacterial biofilms in ocular infections. DNA Cell Biol..

[13] Okajima Y, Kobayakawa S, Tsuji A, Tochikubo T (2006). Biofilm formation by Staphylococcus epidermidis on intraocular lens material. Invest Ophthalmol Vis Sci..

[14] Kodjikian L, Burillon C, Roques C, Pellon G, Renaud FN, Hartmann D, Freney J (2004). Intraocular lenses, bacterial adhesion and endophthalmitis prevention: a review. Biomed Mater Eng..

[15] García-Sáenz MC, Arias-Puente A, Fresnadillo-Martinez MJ, Matilla-Rodriguez A (2000). In vitro adhesion of Staphylococcus epidermidis to intraocular lenses. J Cataract. Refract Surg..

[16] Fey PD, Olson ME (2010). Current concepts in biofilm formation of Staphylococcus epidermidis. Future Microbiol..

[17] Pitts B, Hamilton MA, Zelver N, Stewart PS (2003). A microtiter-plate screening method for biofilm disinfection and removal. J Microbiol Methods..

[18] Arciola CR, Baldassarri L, Montanaro L (2001). Presence of icaA and icaD genes and slime production in a collection of staphylococcal strains from catheter-associated infections. J Clin Microbiol..

[19] Stewart PS, Costerton JW (2001;14). Antibiotic resistance of bacteria in biofilms. Lancet..

[20] Kivanc SA, Akova Budak B, Yildiz M, Kivanc M (2015). The effect of the linezolid and the vancomycine on biofilm production that formed on two different acrylic hydrophobic intraocular lenses. Invest Ophthalmol Vis Sci..

[21] Kodjikian L, Burillon C, Lina G, Roques C, Pellon G, Freney J, Renaud FN (2003). Biofilm formation on intraocular lenses by a clinical strain encoding the ica locus: a scanning electron microscopy study. Invest Ophthalmol Vis Sci..

[22] Prasad S, Nayak N, Satpathy G, Nag TC, Venkatesh P, Pandey RM (2014). Biofilm: The Haven for Staphylococcus epidermidis in Post-operative Endophthalmitis. J Clin Exp Ophthalmol..

[23] Schroeder AC, Schmidbauer JM, Sobke A, Seitz B, Ruprecht KW, Herrmann M (2008). Influence of fibronectin on the adherence of Staphylococcus epidermidis to coated and uncoated intraocular lenses. J Cataract Refract Surg..

[24] Baillif S, Ecochard R, Casoli E, Freney J, Burillon C, Kodjikian L (2008). Adherence and kinetics of biofilm formation of Staphylococcus epidermidis to different types of intraocular lenses under dynamic flow conditions. J Cataract Refract Surg..

[25] Fazly Bazzaz BS, Jalalzadeh M, Sanati M, Zarei-Ghanavati S, Khameneh B (2014). Biofilm Formation by Staphylococcus epidermidis on Foldable and Rigid Intraocular Lenses. Jundishapur J Microbiol..

[26] Vafidis GC, Marsh RJ, Stacey AR (1984). Bacterial contamination of intraocular lens surgery. Br J Ophthalmol..

[27] Bausz M, Fodor E, Resch MD, Kristóf K (2006). Bacterial contamination in the anterior chamber after povidone-iodine application and the effect of the lens implantation device. J Cataract Refract Surg..

[28] Weston K, Nicholson R, Bunce C, Yang YF (2015). An 8-year retrospective study of cataract surgery and postoperative endophthalmitis: injectable intraocular lenses may reduce the incidence of postoperative endophthalmitis. Br J Ophthalmol..

[29] Kıvanç SA, Kıvanç M, Bayramlar H (2016). Microbiology of corneal wounds after cataract surgery: biofilm formation and antibiotic resistance patterns. J Wound Care..

[30] Nomura S, Lundberg F, Stollenwerk M, Nakamura K, Ljungh A (1997). Adhesion of staphylococci to polymers with and without immobilized heparin in cerebrospinal fluid. J Biomed Mater Res..

[31] Cağavi F, Akalan N, Celik H, Gür D, Guçiz B (2004). Effect of hydrophilic coating on microorganism colonization in silicone tubing. Acta Neurochir (Wien)..

[32] Huang XD, Yao K, Zhang H, Huang XJ, Xu ZK (2007). Surface modification of silicone intraocular lens by 2-methacryloyloxyethyl phosphoryl-choline binding to reduce Staphylococcus epidermidis adherence. Clin Exp Ophthalmol..

